# Novel Loci Associated with Increased Risk of Sudden Cardiac Death in the Context of Coronary Artery Disease

**DOI:** 10.1371/journal.pone.0059905

**Published:** 2013-04-04

**Authors:** Adriana Huertas-Vazquez, Christopher P. Nelson, Xiuqing Guo, Kyndaron Reinier, Audrey Uy-Evanado, Carmen Teodorescu, Jo Ayala, Katherine Jerger, Harpriya Chugh, WTCCC+, Peter S. Braund, Panos Deloukas, Alistair S. Hall, Anthony J. Balmforth, Michelle Jones, Kent D. Taylor, Sara L. Pulit, Christopher Newton-Cheh, Karen Gunson, Jonathan Jui, Jerome I. Rotter, Christine M. Albert, Nilesh J. Samani, Sumeet S. Chugh

**Affiliations:** 1 The Heart Institute, Cedars-Sinai Medical Center, Los Angeles, California, United States of America; 2 Department of Cardiovascular Sciences, University of Leicester, Leicester, United Kingdom; 3 NIHR Leicester Cardiovascular Biomedical Research Unit in Cardiovascular Disease, Glenfield Hospital, Leicester, United Kingdom; 4 Medical Genetics Institute, Cedars-Sinai Medical Center, Los Angeles, California, United States of America; 5 Wellcome Trust Sanger Institute, Hinxton, United Kingdom; 6 Department of Clinical Cardiology, University of Leeds, Leeds, United Kingdom; 7 Division of Endocrinology and Metabolism, Department of Medicine, Cedars-Sinai Medical Center, Los Angeles, California, United States of America; 8 Cardiolovascular Research Center and Center for Human Genetic Research, Massachusetts General Hospital, Boston, Massachusetts, United States of America; 9 Pathology, Oregon Health and Science University, Portland, Oregon, United States of America; 10 Emergency Medicine, Oregon Health and Science University, Portland, Oregon, United States of America; 11 Center for Arrhythmia Prevention, Division of Preventive Medicine, Cardiovascular Division, Department of Medicine, Brigham and Women’s Hospital, Harvard Medical School, Boston, Massachusetts, United States of America; Innsbruck Medical University, Austria

## Abstract

**Background:**

Recent genome-wide association studies (GWAS) have identified novel loci associated with sudden cardiac death (SCD). Despite this progress, identified DNA variants account for a relatively small portion of overall SCD risk, suggesting that additional loci contributing to SCD susceptibility await discovery. The objective of this study was to identify novel DNA variation associated with SCD in the context of coronary artery disease (CAD).

**Methods and Findings:**

Using the MetaboChip custom array we conducted a case-control association analysis of 119,117 SNPs in 948 SCD cases (with underlying CAD) from the Oregon Sudden Unexpected Death Study (Oregon-SUDS) and 3,050 controls with CAD from the Wellcome Trust Case-Control Consortium (WTCCC). Two newly identified loci were significantly associated with increased risk of SCD after correction for multiple comparisons at: rs6730157 in the *RAB3GAP1* gene on chromosome 2 (*P* = 4.93×10^−12^, OR = 1.60) and rs2077316 in the *ZNF365* gene on chromosome 10 (P = 3.64×10^−8^, OR = 2.41).

**Conclusions:**

Our findings suggest that *RAB3GAP1* and *ZNF365* are relevant candidate genes for SCD and will contribute to the mechanistic understanding of SCD susceptibility.

## Introduction

Sudden cardiac death (SCD) remains a significant public health problem with an estimated annual incidence of 250,000–300,000 in the US and 4–5 million around the globe [1]–[3]. Although coronary artery disease (CAD) underlies the majority of SCD [4], there is a significant familial component to SCD risk which appears to be distinct from that associated with other manifestations of atherosclerosis in population-based studies [5]–[7]. Recent collaborative genome-wide association (GWA) efforts have identified susceptibility loci associated with SCD [8]–[10] but only two DNA variants on chromosomes 2q24 (*BAZ2B*) [10] and 21q21 (near *CXADR*) [9] have crossed the stringent threshold of genome-wide statistical significance. While candidate-gene based studies have also yielded DNA variants associated with SCD these may not constitute an unbiased approach [11]–[13]. We hypothesized that the distinct configuration of the MetaboChip custom array which contains variants nominally associated (P<0.01) with CAD, QT interval, systolic and diastolic blood pressure, diabetes, glycemic traits, lipids, height and weight in large-scale meta-analyses of GWA studies [14] would enable the identification of additional novel genetic variation associated with SCD in the context of CAD. Accordingly, we conducted a case-control association study using SCD cases from the Oregon Sudden Unexpected Death Study (Oregon-SUDS) and controls with CAD from the Wellcome Trust Case-Control Consortium (WTCCC+).

## Methods

### Study Subjects

#### Ethics statement

All samples have been established in accordance with the principles expressed in the Declaration of Helsinki. The study was approved by the Institutional Review Boards of Oregon Health and Science University, Legacy Health Systems, VA Medical Center, Portland OR and the WTCCC Data Access Committee. Written informed consent was obtained from all enrolled subjects. If subjects were deceased at the time of ascertainment (i.e. following a sudden cardiac death) consent was waived by the respective Institutional Review Boards on grounds of scientific feasibility. These latter subjects were de-identified for the purpose of analysis, in conformation with procedures approved by the respective Institutional Review Boards.

#### Oregon-SUDS

A total of 979 SCD cases of European descent were ascertained from the Oregon-SUDS, an ongoing community-based study among residents of the Portland, Oregon metropolitan area. Detailed methodology has been reported previously [15]–[18]. Briefly, SCD cases are identified from the emergency medical response system, the medical examiner network and 16 local hospitals. All available medical records are obtained for each subject. SCD was defined as an unexpected pulseless condition likely of cardiac origin. If unwitnessed, SCD was defined as unexpected death within 24 hours of having last been seen alive and in normal state of health [19]. All SCD cases included in this study were required to have documented coronary artery disease (CAD). CAD was defined as 50% stenosis of a major coronary artery, physician report of past myocardial infarction (MI), history of percutaneous coronary intervention (PCI) or coronary artery bypass grafting (CABG); or autopsy-identified CAD; or MI by clinical data with any two of the following three: ischemic symptoms, positive troponins or CKMB; or pathologic Q waves on ECG. SCD cases with chronic terminal illnesses, known non-cardiac causes of SCD, traumatic deaths and drug overdose were excluded from the analysis.

#### WTCCC+ controls

The control samples for this study comprised 3,219 pooled subjects from 4 UK studies: (i) CAD cases recruited into the British Heart Foundation Family Heart Study [20] (n = 2169, 78.6% males, 70.8% MI, 68.6% CABG/PCI, mean age at diagnosis 49.9±7.7 years), including the 1,926 subjects analyzed in the original WTCCC Study [21]; (ii) Young MI cases with an event below the age of 50 years recruited into the Premature Acute Myocardial Infarction Study (PRAMIS) [22] (n = 214, 85.5% males, mean age at event 42.4±5.8 years); (iii) MI cases recruited into the Secondary Prevention of Acute Coronary Events - Reduction of Cholesterol to Key European Targets (SPACE ROCKET) Trial [23] (n = 499, 84.0% males, mean age at event 57.7±8.4 years); (iv) MI cases recruited in the Outcomes from Percutaneous coronary intervention by Evaluation of Risk Attributes (OPERA) Trial [24] (n = 337, 75.5% males, mean age at event 55.8±8.4 years). CAD status and diagnosis was validated in all studies by direct review of clinical notes. All subjects were of White European origin. The choice of CAD controls (as opposed to population-based controls) was based on the recognition that over 80% of SCDs occur in the setting of CAD. Accordingly, CAD controls would enable the discovery of genetic associations exclusively related to SCD and independent of CAD [25].

### MetaboChip Array

The MetaboChip array (Illumina, San Diego, CA) is a custom Illumina iSelect genotyping array comprised of approximately 200,000 SNPs selected from previous GWAS meta-analyses findings from the CARDIoGRAM (coronary artery disease), DIAGRAM (type 2 diabetes), GIANT (height and weight), MAGIC (glycemic traits), Lipids (lipids), ICBP-GWAS (blood pressure), and QT-IGC (QT interval) consortia [14]. The array comprises a linkage-disequilibrium pruned set of SNPs that reached a nominal level (P<0.01) of association with each of these diseases/phenotypes as well as SNP sets for fine-mapping of loci identified for these disease/traits at the time of design of the array. Additional details of the MetaboChip design can be found at: www.sph.umich.edu/csg/kang/MetaboChip/.

### MetaboChip Genotyping and Statistical Analysis

Genotyping of SCD cases was performed at the Medical Genetics Institute at Cedars-Sinai Medical Center, Los Angeles, U.S. and controls were genotyped at the Wellcome Trust Sanger Centre, Hinxton, UK using the same array. After exclusion of array failures, poor quality genotypes and duplicates, 948 SCD cases and 3,050 CAD controls were used in the current analyses.

Genotypes in both studies were called using the GenCall algorithm [26], [27]. Individual SNPs were excluded from analysis using standard quality control criteria based on sample call rates less than 90%, Hardy-Weinberg Equilibrium (HWE) P<1×10^−4^, monomorphic and SNPs with minor allele frequencies (MAF) less than 1%. This left 119,117 post-QC SNPs for analysis. Association analyses were performed using logistic regression assuming an additive model adjusting for age, sex and the first 3 dimensions from multi-dimensional scaling (PLINK software) [28]. Results were further corrected for the genomic control factor (λ), which was calculated after excluding SNPs related to QT interval and CAD.

## Results

**Figure 1:**
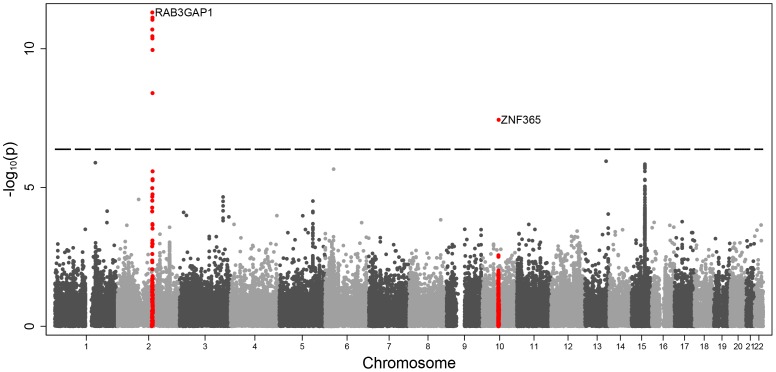
Manhattan plot of associated findings. Data is displayed as –log_10_P values against chromosomal location for the 119,117 SNPs that were included in the statistical analysis. The dotted line represents the conservative significance threshold of P = 4.2×10^−7^. The two loci that showed an association at this level are plotted in red.

The mean ages of the subjects in the Oregon-SUDS and the WTCCC studies were 60.8±12.6 and 51.4±7.5 years respectively at time of event. Seventy two percent of SCD cases and 80.9% of CAD controls were male. The genomic control factor (λ) for this analysis was 1.25. Based on the number of SNPs tested (119,117 SNPs) a significance *P*-value cut off of 4.2×10^−7^ was determined. This level of correction for multiple testing is probably conservative given that the MetaboChip array contains many DNA variants in strong linkage disequilibrium, especially within the fine-mapping sets. Nonetheless, we observed SNPs exceeding the array-wide significance threshold (*P* = 4.2×10^−7^) as well as genome-wide significance (P<5×10^−8^) after correcting for lambda inflation in two loci on chromosomes: 2q21 and 10q21 (Figure 1). The association results of the lead variants at each of these loci are shown in Table 1. The strongest associations were observed for the intronic SNPs rs6730157 (*P* = 4.93×10^−12^, OR = 1.60, 95%) within *RAB3GAP*1 and rs2077316 in the ZNF365 gene (*P* = 3.64×10^−8^, OR = 2.41).

**Table 1:** Summary of the two loci associated with SCD.

Chr	SNP	Gene	Position	Associatedallele	Associated allele frequency SCD cases	Associated allele frequency CAD controls	SE	OR	λ Corrected P-value
2	rs6730157	*RAB3GAP1*	135623558	G	0.37	0.26	0.06	1.60	4.93×10^−12^
10	rs2077316	*ZNF365*	63895454	C	0.060	0.026	0.14	2.41	3.64×10^−8^

Chr, chromosome; SE, standard error; OR, odds ratio.

**Figure 2:**
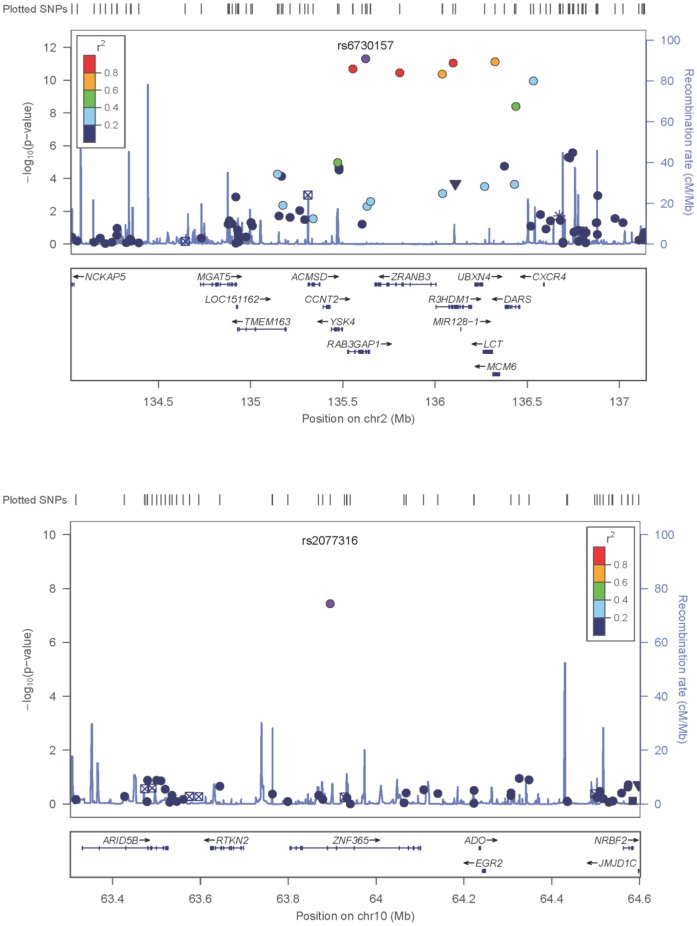
Regional association plots for the two associated SNPs with SCD. Each SNP is plotted with respect to its chromosomal location (x-axis) and –log_10_P-value (y-axis on the left). The spikes indicate the recombination rate (y-axis on the right) at that region of the chromosome.

The association signal on 2q21 spans quite a large region with multiple SNPs showing a significant association (Figure 1). In conditional analysis, rs6730157 remained the most significant SNP and no other SNP in the region had a significant association (P<0.01). Figure 2 shows the regional association plots for rs6730157 and rs2077316.

## Discussion

We have conducted a case-control association study using the MetaboChip array to identify novel genetic variation associated with SCD independent of CAD. The most significantly associated SNP (rs6730157, P = 4.93×10^−12^) resides in an intronic region of the *RAB3GAP1* gene on chromosome 2q21. *RAB3GAP1* encodes the catalytic subunit of RabGTPase activating protein. *RAB3GAP*, which is involved in regulation of *RAB3* activity, is a heterodimeric complex consisting of a 130-kD catalytic subunit. Mutations in *RAB3GAP1* are associated with Warburg micro syndrome, a rare autosomal recessive syndrome characterized by microcephaly, severe mental retardation and cataracts [29]. *RAB3GAP1* is a key regulator of calcium mediated hormone and neurotransmitter exocytosis [30], [31]. Interestingly, a previous study performed in a yeast two-hybrid system and a rat dorsal root ganglion found that a protein similar to human *RAB3GAP1* interacts with intracellular domains of *SCN10A* [32]. DNA variation within *SCN10A* has been associated with abnormalities of cardiac ventricular depolarization, conduction, and ventricular fibrillation [33]–[36].

To test whether rs6730157 was located in a regulatory region or transcription factor binding domain, we searched the ENCODE project (Encyclopedia of DNA elements) database. We found that rs6730157 is predicted to fall into a strong enhancer in several cell types, including cardiac and aortic adventitial fibroblast cells [37]. However, it should be noted that although *RAB3GAP1* is a strong candidate gene in the chromosome 2 locus, the association signal spans several others genes (Figure 2). At this stage, in common with other GWAS findings, we cannot exclude the possibility that the association is driven by another gene at this locus. Fine mapping and functional analysis of the locus will be required to refine the association.

The second significantly associated SNP (rs2077316, P = 3.64×10^−8^) resides in an intronic region of the zinc finger protein 365 gene (*ZNF365*) on chromosome 10q21. *ZNF365* encodes several isoforms which have different expression patterns and functions. *ZNF365* has been implicated in breast cancer [38] and Crohn’s disease [39] and a role in heart disease has not been reported. According to ENCODE, no regulatory effects for rs2077316 are currently predicted [37].

Our study has several limitations. Despite attempting to take any population stratification into account using multi-dimensional scaling, we observed an inflation of the genomic control factor statistic (λ). This could be due to further differences in population structure between the SCD cases and CAD controls which, while all of European descent, are drawn from individuals from two separate countries. Alternately, the design of the MetaboChip with a possible over-representation of variants of relevance given the choices of traits used to select the SNPs could contribute to an inflation of this statistic. We tried to limit the impact of this by excluding SNPs related to QT interval and CAD when calculating the genomic control factor statistic. Most importantly, our findings currently lack replication. In this context, although the association at the 2q21 locus looks robust (with the association exceeding GWA significance by several log values), particular caution needs to be exercised in the interpretation of the finding at 10q21 as only a single SNP with a very low minor allele frequency (Table 1) showed an association. Replication of the findings is challenging because of the rarity of collections of SCD subjects occurring in the context of CAD. Nonetheless, in both cases our findings should be considered provisional until further corroboration.

In summary, we provide evidence for two novel loci where variants may affect risk of SCD in the context of CAD. Understanding the mechanisms that increase risk of SCD is an essential first step in trying to reduce this important complication of CAD.

## Supporting Information

File S1 **The full list of WTCCC+ members.**
(DOC)

## References

[1] Chugh SS, Reinier K, Teodorescu C, Evanado A, Kehr E, et al (2008) Epidemiology of sudden cardiac death: Clinical and research implications. Prog Cardiovasc Dis 51: 213–228. 19026856 10.1016/j.pcad.2008.06.003 PMC2621010

[2] Adabag AS, Luepker RV, Roger VL, Gersh BJ (2010) Sudden cardiac death: Epidemiology and risk factors. Nat Rev Cardiol 7: 216–225. 20142817 10.1038/nrcardio.2010.3 PMC5014372

[3] Fishman GI, Chugh SS, DiMarco J, Albert C, Anderson M, et al (2010) Sudden Cardiac Death Prediction and Prevention Report from a National Heart, Lung, and Blood Institute and Heart Rhythm Society Workshop. Circulation 122: 2335–2348. 21147730 10.1161/CIRCULATIONAHA.110.976092 PMC3016224

[4] Myerburg RJ, Junttila MJ (2012) Sudden cardiac death caused by coronary heart disease. Circulation 125: 1043–1052. 22371442 10.1161/CIRCULATIONAHA.111.023846

[5] Jouven X, Desnos M, Guerot C, Ducimetiere P (1999) Predicting sudden death in the population: The Paris Prospective Study I. Circulation. 99: 1978–1983. 10.1161/01.cir.99.15.1978 10209001

[6] Friedlander Y, Siscovick DS, Arbogast P, Psaty BM, Weinmann S, et al (2002) Sudden death and myocardial infarction in first degree relatives as predictors of primary cardiac arrest. Atherosclerosis 162: 211–216. 11947916 10.1016/s0021-9150(01)00701-8

[7] Kaikkonen KS, Kortelainen ML, Linna E, Huikuri HV (2006) Family history and the risk of sudden cardiac death as a manifestation of an acute coronary event. Circulation 114: 1462–1467. 17000909 10.1161/CIRCULATIONAHA.106.624593

[8] Arking DE, Reinier K, Post W, Jui J, Hilton G, et al (2010) Genome-wide association study identifies GPC5 as a novel genetic locus protective against sudden cardiac arrest. PLoS One 5: e9879. 20360844 10.1371/journal.pone.0009879 PMC2845611

[9] Bezzina CR, Pazoki R, Bardai A, Marsman RF, de Jong JS, et al (2010) Genome-wide association study identifies a susceptibility locus at 21q21 for ventricular fibrillation in acute myocardial infarction. Nat Genet 42: 688–691. 20622880 10.1038/ng.623 PMC3966292

[10] Arking DE, Junttila MJ, Goyette P, Huertas-Vazquez A, Eijgelsheim M, et al (2011) Identification of a sudden cardiac death susceptibility locus at 2q24.2 through genome-wide association in European ancestry individuals. PLoS Genet 7: e1002158. 21738491 10.1371/journal.pgen.1002158 PMC3128111

[11] Arking D, Chugh SS, Chakravarti A, Spooner PM (2004) Genomics in Sudden Cardiac Death. Circ Res 94: 712–723. 15059941 10.1161/01.RES.0000123861.16082.95

[12] Westaway SK, Reinier K, Huertas-Vazquez A, Evanado A, Teodorescu C, et al (2011) Common variants in CASQ2, GPD1L, and NOS1AP are significantly associated with risk of sudden death in patients with coronary artery disease. Circ Cardiovasc Genet 4: 397–402. 21685173 10.1161/CIRCGENETICS.111.959916 PMC3160237

[13] Albert CM, MacRae CA, Chasman DI, VanDenburgh M, Buring JE, et al (2010) Common variants in cardiac ion channel genes are associated with sudden cardiac death. Circ Arrhythm Electrophysiol 3: 222–229. 20400777 10.1161/CIRCEP.110.944934 PMC2891421

[14] Voight BF, Kang HM, Ding J, Palmer CD, Sidore C, et al (2012) The metabochip, a custom genotyping array for genetic studies of metabolic, cardiovascular, and anthropometric traits. PLoS Genet 8: e1002793. 22876189 10.1371/journal.pgen.1002793 PMC3410907

[15] Chugh SS, Jui J, Gunson K, Stecker EC, John BT, et al (2004) Current burden of sudden cardiac death: multiple source surveillance versus retrospective death certificate-based review in a large U.S. community. J Am Coll Cardiol 44: 1268–1275. 15364331 10.1016/j.jacc.2004.06.029

[16] Stecker EC, Vickers C, Waltz J, Socoteanu C, John BT, et al (2006) Population-based analysis of sudden cardiac death with and without left ventricular systolic dysfunction: two-year findings from the Oregon Sudden Unexpected Death Study. J Am Coll Cardiol 47: 1161–1166. 16545646 10.1016/j.jacc.2005.11.045

[17] Chugh SS, Uy-Evanado A, Teodorescu C, Reinier K, Mariani R, et al (2009) Women have a lower prevalence of structural heart disease as a precursor to sudden cardiac arrest: The Ore-SUDS (Oregon Sudden Unexpected Death Study). J Am Coll Cardiol 54: 2006–2011. 19926005 10.1016/j.jacc.2009.07.038 PMC2850557

[18] Chugh SS, Reinier K, Singh T, Uy-Evanado A, Socoteanu C, et al (2009) Determinants of prolonged QT interval and their contribution to sudden death risk in coronary artery disease: the Oregon Sudden Unexpected Death Study. Circulation 119: 663–670. 19171855 10.1161/CIRCULATIONAHA.108.797035 PMC2734945

[19] World Health Organization (1985) Sudden cardiac death. Report of a WHO Scientific Group. World Health Organ Tech Rep Ser 726: 5–25. 3936284

[20] Samani NJ, Burton P, Mangino M, Ball SG, Balmforth AJ, et al (2005) A genomewide linkage study of 1,933 families affected by premature coronary artery disease: The British Heart Foundation (BHF) Family Heart Study. Am J Hum Genet 77: 1011–1020. 16380912 10.1086/498653 PMC1285159

[21] Wellcome Trust Case Control C (2007) Genome-wide association study of 14,000 cases of seven common diseases and 3,000 shared controls. Nature 447: 661–678. 17554300 10.1038/nature05911 PMC2719288

[22] Brouilette S, Singh RK, Thompson JR, Goodall AH, Samani NJ (2003) White cell telomere length and risk of premature myocardial infarction. Arterioscler Thromb Vasc Biol 23: 842–846. 12649083 10.1161/01.ATV.0000067426.96344.32

[23] Hall AS, Jackson BM, Farrin AJ, Efthymiou M, Barth JH, et al (2009) A randomized, controlled trial of simvastatin versus rosuvastatin in patients with acute myocardial infarction: the Secondary Prevention of Acute Coronary Events-Reduction of Cholesterol to Key European Targets Trial. Eur J Cardiovasc Prev Rehabil 16: 712–721. 19745745 10.1097/HJR.0b013e3283316ce8

[24] Pearson IR, Sivananthan UM, Barth JH, Gale CP, Hall AS (2011) Comparison of 4-H Heart Fatty Acid Binding Protein with 12-H Troponin I to Assess 6-Month Risk Following Percutaneous Coronary Intervention in Acute Coronary Syndromes. Heart 97: A17–A17.

[25] Adabag AS, Peterson G, Apple FS, Titus J, King R, et al (2010) Etiology of sudden death in the community: results of anatomical, metabolic, and genetic evaluation. Am heart J 159: 33–39. 20102864 10.1016/j.ahj.2009.10.019 PMC2905235

[26] Illumina Inc. (2005) Illumina GenCall Data Analysis Software. *Technology Spotlight* Available: http://www.illumina.com/Documents/products/technotes/technote_gencall_data_analysis_software.pdf. Accessed 2013 Jan.

[27] Illumina Inc. (2009) Improved Cluster Generation with Gentrain2. *Technical Note: DNA Analysis* Available: http://www.illumina.com/Documents/products%5Ctechnotes%5Ctechnote_gentrain2.pdf. Accessed 2013 Jan.

[28] Purcell S, Neale B, Todd-Brown K, Thomas L, Ferreira MAR, et al (2007) PLINK: A tool set for whole-genome association and population-based linkage analyses. Am J Hum Genet 81: 559–575. 17701901 10.1086/519795 PMC1950838

[29] Aligianis IA, Johnson CA, Gissen P, Chen D, Hampshire D, et al (2005) Mutations of the catalytic subunit of RAB3GAP cause Warburg Micro syndrome. Nat Genet 37: 221–223. 15696165 10.1038/ng1517

[30] Takai Y, Sasaki T, Shirataki H, Nakanishi H (1996) Rab3A small GTP-binding protein in Ca(2+)-dependent exocytosis. Genes Cells 1: 615–632. 9078389 10.1046/j.1365-2443.1996.00257.x

[31] Aligianis IA, Morgan NV, Mione M, Johnson CA, Rosser E, et al (2006) Mutation in Rab3 GTPase-activating protein (RAB3GAP) noncatalytic subunit in a kindred with Martsolf syndrome. Am J Hum Genet 78: 702–707. 16532399 10.1086/502681 PMC1424696

[32] Malik-Hall M, Poon WY, Baker MD, Wood JN, Okuse K (2003) Sensory neuron proteins interact with the intracellular domains of sodium channel NaV1.8. Brain Res Mol Brain Res 110: 298–304. 12591166 10.1016/s0169-328x(02)00661-7

[33] Sotoodehnia N, Isaacs A, de Bakker PI, Dorr M, Newton-Cheh C, et al (2010) Common variants in 22 loci are associated with QRS duration and cardiac ventricular conduction. Nat Genet 42: 1068–1076. 21076409 10.1038/ng.716 PMC3338195

[34] Pfeufer A, van Noord C, Marciante KD, Arking DE, Larson MG, et al (2010) Genome-wide association study of PR interval. Nat Genet 42: 153–159. 20062060 10.1038/ng.517 PMC2850197

[35] Chambers JC, Zhao J, Terracciano CM, Bezzina CR, Zhang W, et al (2010) Genetic variation in SCN10A influences cardiac conduction. Nat Genet 42: 149–152. 20062061 10.1038/ng.516

[36] Holm H, Gudbjartsson DF, Arnar DO, Thorleifsson G, Thorgeirsson G, et al (2010) Several common variants modulate heart rate, PR interval and QRS duration. Nat Genet 42: 117–122. 20062063 10.1038/ng.511

[37] Ward LD, Kellis M (2012) HaploReg: a resource for exploring chromatin states, conservation, and regulatory motif alterations within sets of genetically linked variants. Nucleic Acids Res 40: D930–934. 22064851 10.1093/nar/gkr917 PMC3245002

[38] Turnbull C, Ahmed S, Morrison J, Pernet D, Renwick A, et al (2010) Genome-wide association study identifies five new breast cancer susceptibility loci. Nat Genet 42: 504–507. 20453838 10.1038/ng.586 PMC3632836

[39] Barrett JC, Hansoul S, Nicolae DL, Cho JH, Duerr RH, et al (2008) Genome-wide association defines more than 30 distinct susceptibility loci for Crohn’s disease. Nat Genet 40: 955–962. 18587394 10.1038/NG.175 PMC2574810

