# Supplementary material for: Novel Loci Associated with Increased Risk of Sudden Cardiac Death in the Context of Coronary Artery Disease
Source: PLoS One. 2013 Apr 4;8(4):e59905. doi: 10.1371/journal.pone.0059905 (PMC3617189; doi:10.1371/journal.pone.0059905)
Supplement: File S1 — The full list of WTCCC+ members. (DOC) [file pone.0059905.s001.doc]

**Members of the WTCCC+ consortium (Dec 2011)**

Jan Aerts^1^, Tariq Ahmad^2^, Hazel Arbury^1^, Anthony Attwood^1,3,4^, Adam Auton^5^, Stephen G Ball^6^, Anthony J Balmforth^6^, Chris Barnes^1^, Jeffrey C Barrett^1^, Inês Barroso^1^, Anne Barton^7^, Amanda J Bennett^8^, Sanjeev Bhaskar^1^, Katarzyna Blaszczyk^9^, John Bowes^7^, Oliver J Brand^8,10^, Peter S Braund^11^, Francesca Bredin^12^, Gerome Breen^13,14^, Morris J Brown^15^, Ian N Bruce^7^, Jaswinder Bull^16^, Oliver S Burren^17^, John Burton^1^, Jake Byrnes^18^, Sian Caesar^19^, Niall Cardin^5^, Chris M Clee^1^, Alison J Coffey^1^, John MC Connell^20^, Donald F Conrad^1^, Jason D Cooper^17^, Anna F Dominiczak^20^, Kate Downes^17^, Hazel E Drummond^21^, Darshna Dudakia^16^, Andrew Dunham^1^, Bernadette Ebbs^16^, Diana Eccles^22^, Sarah Edkins^1^, Cathryn Edwards^23^, Anna Elliot^16^, Paul Emery^24^, David M Evans^25^, Gareth Evans^26^, Steve Eyre^7^, Anne Farmer^14^, I Nicol Ferrier^27^, Edward Flynn^7^, Alistair Forbes^28^, Liz Forty^29^, Jayne A Franklyn^10,30^, Timothy M Frayling^2^, Rachel M Freathy^2^, Eleni Giannoulatou^5^, Polly Gibbs^16^, Paul Gilbert^7^, Katherine Gordon-Smith^19,29^, Emma Gray^1^, Elaine Green^29^, Chris J Groves^8^, Detelina Grozeva^29^, Rhian Gwilliam^1^, Anita Hall^16^, Naomi Hammond^1^, Matt Hardy^17^, Pile Harrison^31^, Neelam Hassanali^8^, Husam Hebaishi^1^, Sarah Hines^16^, Anne Hinks^7^, Graham A Hitman^32^, Lynne Hocking^33^, Chris Holmes^5^, Eleanor Howard^1^, Philip Howard^34^, Joanna MM Howson^17^, Debbie Hughes^16^, Sarah Hunt^1^, John D Isaacs^35^, Mahim Jain^18^, Derek P Jewell^36^, Toby Johnson^34^, Jennifer D Jolley^3,4^, Ian R Jones^29^, Lisa A Jones^19^, George Kirov^29^, Cordelia F Langford^1^, Hana Lango-Allen^2^, G Mark Lathrop^37^, James Lee^12^, Kate L Lee^34^, Charlie Lees^21^, Kevin Lewis^1^, Cecilia M Lindgren^8,18^, Meeta Maisuria-Armer^17^, Julian Maller^18^, John Mansfield^38^, Jonathan L Marchini^5^, Paul Martin^7^, Dunecan CO Massey^12^, Wendy L McArdle^39^, Peter McGuffin^14^, Kirsten E McLay^1^, Gil McVean^5,18^, Alex Mentzer^40^, Michael L Mimmack^1^, Ann E Morgan^41^, Andrew P Morris^18^, Craig Mowat^42^, Patricia B Munroe^34^, Simon Myers^18^, William Newman^26^, Elaine R Nimmo^21^, Michael C O'Donovan^29^, Abiodun Onipinla^34^, Nigel R Ovington^17^, Michael J Owen^29^, Kimmo Palin^1^, Aarno Palotie^1^, Kirstie Parnell^2^, Richard Pearson^8^, David Pernet^16^, John RB Perry^2,18^, Anne Phillips^42^, Vincent Plagnol^17^, Natalie J Prescott^9^, Inga Prokopenko^8,18^, Michael A Quail^1^, Suzanne Rafelt^11^, Nigel W Rayner^8,18^, David M Reid^33^, Anthony Renwick^16^, Susan M Ring^39^, Neil Robertson^8,18^, Samuel Robson^1^, Ellie Russell^29^, David St Clair^13^, Jennifer G Sambrook^3,4^, Jeremy D Sanderson^40^, Stephen J Sawcer^43^, Helen Schuilenburg^17^, Carol E Scott^1^, Richard Scott^16^, Sheila Seal^16^, Sue Shaw-Hawkins^34^, Beverley M Shields^2^, Matthew J Simmonds^8,10^, Debbie J Smyth^17^, Elilan Somaskantharajah^1^, Katarina Spanova^16^, Sophia Steer^44^, Jonathan Stephens^3,4^, Helen E Stevens^17^, Kathy Stirrups^1^, Millicent A Stone^45,46^, David P Strachan^47^, Zhan Su^5^, Deborah PM Symmons^7^, John R Thompson^48^, Wendy Thomson^7^, Martin D Tobin^48^, Mary E Travers^8^, Clare Turnbull^16^, Damjan Vukcevic^18^, Louise V Wain^48^, Mark Walker^49^, Neil M Walker^17^, Chris Wallace^17^, Margaret Warren-Perry^16^, Nicholas A Watkins^3,4^, John Webster^50^, Michael N Weedon^2^, Anthony G Wilson^51^, Matthew Woodburn^17^, B Paul Wordsworth^52^, Chris Yau^5^, Allan H Young^27,53^, Eleftheria Zeggini^1^, Matthew A Brown^52,54^, Paul R Burton^48^, Mark J Caulfield^34^, Alastair Compston^43^, Martin Farrall^55^, Stephen CL Gough^8,10,30^, Alistair S Hall^6^, Andrew T Hattersley^2,56^, Adrian VS Hill^18^, Christopher G Mathew^9^, Marcus Pembrey^57^, Jack Satsangi^21^, Michael R Stratton^1,16^, Jane Worthington^7^, Matthew E Hurles^1^, Audrey Duncanson^58^, Willem H Ouwehand^1,3,4^, Miles Parkes^12^, Nazneen Rahman^16^, John A Todd^17^, Nilesh J Samani^11,59^, Dominic P Kwiatkowski^1,18^, Mark I McCarthy^8,18,60^, Nick Craddock^29^, Panos Deloukas^1^, Peter Donnelly^5,18^.

^1^ The Wellcome Trust Sanger Institute, Wellcome Trust Genome Campus, Hinxton, Cambridge, CB10 1SA UK.

^2^ Genetics of Complex Traits, Peninsula College of Medicine and Dentistry University of Exeter, EX1 2LU, UK.

^3^ Department of Haematology, University of Cambridge, Long Road, Cambridge, CB2 0PT, UK.

^4^ National Health Service Blood and Transplant, Cambridge Centre, Long Road, Cambridge CB2 0PT, UK.

^5^ Department of Statistics, University of Oxford, 1 South Parks Road, Oxford, OX1 3TG, UK.

^6^ Multidisciplinary Cardiovascular Research Centre (MCRC), Leeds Institute of Genetics, Health and Therapeutics (LIGHT), University of Leeds, Leeds, LS2 9JT, UK.

^7^ arc Epidemiology Unit, Stopford Building, University of Manchester, Oxford Road, Manchester, M13 9PT, UK.

^8^ Oxford Centre for Diabetes, Endocrinology and Medicine, University of Oxford, Churchill Hospital, Oxford OX3 7LJ, UK.

^9^ Department of Medical and Molecular Genetics, King’s College London School of Medicine, 8th Floor Guy’s Tower, Guy’s Hospital, London, SE1 9RT, UK.

^10^ Centre for Endocrinology, Diabetes and Metabolism, Institute of Biomedical Research, University of Birmingham, Birmingham, B15 2TT, UK.

^11^ Department of Cardiovascular Sciences, University of Leicester, Glenfield Hospital, Groby Road, Leicester LE3 9QP, UK.

^12^ IBD Genetics Research Group, Addenbrooke's Hospital, Cambridge, CB2 0QQ, UK.

^13^ University of Aberdeen, Institute of Medical Sciences, Foresterhill, Aberdeen AB25 2ZD, UK.

^14^ SGDP, The Institute of Psychiatry, King's College London, De Crespigny Park, Denmark Hill, London SE5 8AF, UK.

^15^ Clinical Pharmacology Unit, University of Cambridge, Addenbrookes Hospital, Hills Road, Cambridge CB2 2QQ, UK.

^16^ Section of Cancer Genetics, Institute of Cancer Research, 15 Cotswold Road, Sutton SM2 5NG, UK.

^17^  Juvenile Diabetes Research Foundation/Wellcome Trust Diabetes and Inflammation Laboratory, Department of Medical Genetics, Cambridge Institute for Medical Research, University of Cambridge, Wellcome Trust/MRC Building, Cambridge CB2 0XY, UK.

^18^ The Wellcome Trust Centre for Human Genetics, University of Oxford, Roosevelt Drive, Oxford OX3 7BN, UK.

^19^ Department of Psychiatry, University of Birmingham, National Centre for Mental Health, 25 Vincent Drive, Birmingham, B15 2FG, UK.

^20^ BHF Glasgow Cardiovascular Research Centre, University of Glasgow, 126 University Place, Glasgow, G12 8TA, UK.

^21^ Gastrointestinal Unit, Division of Medical Sciences, School of Molecular and Clinical Medicine, University of Edinburgh, Western General Hospital, Edinburgh EH4 2XU, UK.

^22^ Academic Unit of Genetic Medicine, University of Southampton, Southampton, UK.

^23^ Endoscopy Regional Training Unit, Torbay Hospital, Torbay TQ2 7AA, UK.

^24^ Academic Unit of Musculoskeletal Disease, University of Leeds, Chapel Allerton Hospital, Leeds, West Yorkshire LS7 4SA, UK.

^25^ MRC Centre for Causal Analyses in Translational Epidemiology, Department of Social Medicine, University of Bristol, Bristol, BS8 2BN, UK.

^26^ Department of Medical Genetics, Manchester Academic Health Science Centre (MAHSC), University of Manchester, Manchester M13 0JH, UK.

^27^ School of Neurology, Neurobiology and Psychiatry, Royal Victoria Infirmary, Queen Victoria Road, Newcastle upon Tyne, NE1 4LP, UK.

^28^ Institute for Digestive Diseases, University College London Hospitals Trust, London NW1 2BU, UK.

^29^ MRC Centre for Neuropsychiatric Genetics and Genomics, School of Medicine, Cardiff University, Heath Park, Cardiff, CF14 4XN, UK.

^30^ University Hospital Birmingham NHS Foundation Trust, Birmingham, B15 2TT, UK.

^31^ University of Oxford, Institute of Musculoskeletal Sciences, Botnar Research Centre, Oxford, OX3 7LD, UK.

^32^ Centre for Diabetes and Metabolic Medicine, Barts and The London, Royal London Hospital, Whitechapel, London, E1 1BB, UK.

^33^ Bone Research Group, Department of Medicine and Therapeutics, University of Aberdeen, Aberdeen, AB25 2ZD, UK.

^34^ Clinical Pharmacology and Barts and The London Genome Centre, William Harvey Research Institute, Barts and The London School of Medicine and Dentistry, Queen Mary University of London, Charterhouse Square, London EC1M 6BQ, UK.

^35^ Institute of Cellular Medicine, Musculoskeletal Research Group, 4th Floor, Catherine Cookson Building, The Medical School, Framlington Place, Newcastle upon Tyne, NE2 4HH, UK.

^36^ Gastroenterology Unit, Radcliffe Infirmary, University of Oxford, Oxford, OX2 6HE, UK.

^37^  Centre National de Genotypage, 2, Rue Gaston Cremieux, Evry, Paris 91057, France.

^38^ Department of Gastroenterology & Hepatology, University of Newcastle upon Tyne, Royal Victoria Infirmary, Newcastle upon Tyne NE1 4LP, UK.

^39^ ALSPAC Laboratory, Department of Social Medicine, University of Bristol, BS8 2BN, UK.

^40^ Division of Nutritional Sciences, King's College London School of Biomedical and Health Sciences, London SE1 9NH, UK.

^41^ NIHR-Leeds Musculoskeletal Biomedical Research Unit, University of Leeds, Chapel Allerton Hospital, Leeds, West Yorkshire LS7 4SA, UK.

^42^ Department of General Internal Medicine, Ninewells Hospital and Medical School, Ninewells Avenue, Dundee DD1 9SY, UK.

^43^ Department of Clinical Neurosciences, University of Cambridge, Addenbrooke's Hospital, Hills Road, Cambridge, CB2 2QQ, UK.

^44^ Clinical and Academic Rheumatology, Kings College Hosptal National Health Service Foundation Trust, Denmark Hill, London SE5 9RS, UK.

^45^ University of Toronto, St. Michael's Hospital, 30 Bond Street, Toronto, Ontario M5B 1W8, Canada.

^46^ University of Bath, Claverdon, Norwood House, Room 5.11a Bath Somerset BA2 7AY, UK.

^47^ Division of Community Health Sciences, St George's, University of London, London SW17 0RE, UK.

^48^ Departments of Health Sciences and Genetics, University of Leicester, 217 Adrian Building, University Road, Leicester, LE1 7RH, UK.

^49^ Diabetes Research Group, School of Clinical Medical Sciences, Newcastle University, Framlington Place, Newcastle upon Tyne NE2 4HH, UK.

^50^ Medicine and Therapeutics, Aberdeen Royal Infirmary, Foresterhill, Aberdeen, Grampian AB9 2ZB, UK.

^51^ School of Medicine and Biomedical Sciences, University of Sheffield, Sheffield, S10 2JF, UK.

^52^ Nuffield Department of Orthopaedics, Rheumatology and Musculoskeletal Sciences, Nuffield Orthopaedic Centre, University of Oxford, Windmill Road, Headington, Oxford, OX3 7LD, UK.

^53^ UBC Institute of Mental Health, 430-5950 University Boulevard Vancouver, British Columbia, V6T 1Z3, Canada.

^54^ Diamantina Institute of Cancer, Immunology and Metabolic Medicine, Princess Alexandra Hospital, University of Queensland, Ipswich Road, Woolloongabba, Brisbane, Queensland, 4102, Australia.

^55^ Cardiovascular Medicine, University of Oxford, Wellcome Trust Centre for Human Genetics, Roosevelt Drive, Oxford OX3 7BN, UK.

^56^ Genetics of Diabetes, Peninsula College of Medicine and Dentistry, University of Exeter, Barrack Road, Exeter, EX2 5DW, UK.

^57^ Clinical and Molecular Genetics Unit, Institute of Child Health, University College London, 30 Guilford Street, London WC1N 1EH, UK.

^58^ The Wellcome Trust, Gibbs Building, 215 Euston Road, London NW1 2BE, UK.

^59^ Leicester NIHR Biomedical Research Unit in Cardiovascular Disease, Glenfield Hospital, Leicester, LE3 9QP, UK.

^60^ Oxford NIHR Biomedical Research Centre, Churchill Hospital, Oxford, OX3 7LJ, UK.
